# Advances in the Synthesis and Physiological Metabolic Regulation of Nicotinamide Mononucleotide

**DOI:** 10.3390/nu16142354

**Published:** 2024-07-20

**Authors:** Chuxiong Zheng, Yumeng Li, Xin Wu, Le Gao, Xiaoyi Chen

**Affiliations:** 1 School of Biological Engineering, Dalian Polytechnic University, Dalian 116034, China; zhengchuxiong@tib.cas.cn; 2 National Technology Innovation Center for Synthetic Biology, Tianjin Institute of Industrial Biotechnology, Chinese Academy of Sciences, No. 32, Xiqi Road, Tianjin Airport Economic Park, Tianjin 300308, China; liym@tib.cas.cn (Y.L.); wuxin@tib.cas.cn (X.W.)

**Keywords:** NMN, NAD^+^, biosynthesis, cell factories, biological activity

## Abstract

Nicotinamide mononucleotide (NMN), the direct precursor of nicotinamide adenine dinucleotide (NAD^+^), is involved in the regulation of many physiological and metabolic reactions in the body. NMN can indirectly affect cellular metabolic pathways, DNA repair, and senescence, while also being essential for maintaining tissues and dynamic metabolic equilibria, promoting healthy aging. Therefore, NMN has found many applications in the food, pharmaceutical, and cosmetics industries. At present, NMN synthesis strategies mainly include chemical synthesis and biosynthesis. Despite its potential benefits, the commercial production of NMN by organic chemistry approaches faces environmental and safety problems. With the rapid development of synthetic biology, it has become possible to construct microbial cell factories to produce NMN in a cost-effective way. In this review, we summarize the chemical and biosynthetic strategies of NMN, offering an overview of the recent research progress on host selection, chassis cell optimization, mining of key enzymes, metabolic engineering, and adaptive fermentation strategies. In addition, we also review the advances in the role of NMN in aging, metabolic diseases, and neural function. This review provides comprehensive technical guidance for the efficient biosynthesis of NMN as well as a theoretical basis for its application in the fields of food, medicine, and cosmetics.

## 1. Introduction

**Figure 1:**
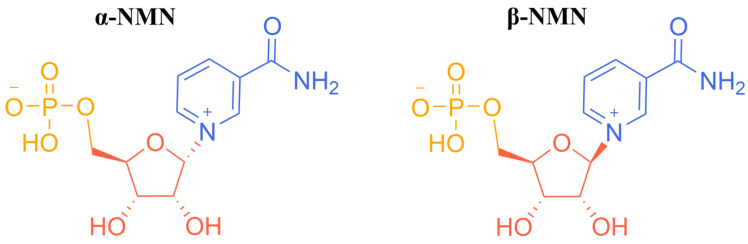
The two N-stereoisomers of NMN.

Nicotinamide mononucleotide (NMN) is a naturally occurring vitamin B derivative that is a bioactive nucleotide with a molecular weight of 334.221 g/mol [1,2]. It exists in α and β forms (Figure 1), but only the latter has biological activity. NMN occurs naturally in some vegetables (0.25–1.88 mg/100 g) and fruits (0.26–1.60 mg/100 g), including edamame, cabbage, cucumber, broccoli, tomato, mushrooms, and avocado [3]. In addition, raw beef and shrimp also contain a small amount of NMN (0.06–0.42 mg/100 g). In vivo, NMN is mainly located in red blood cells, and the amounts required to maintain normal physiological functions can be obtained from the daily diet [4].

**Figure 2:**
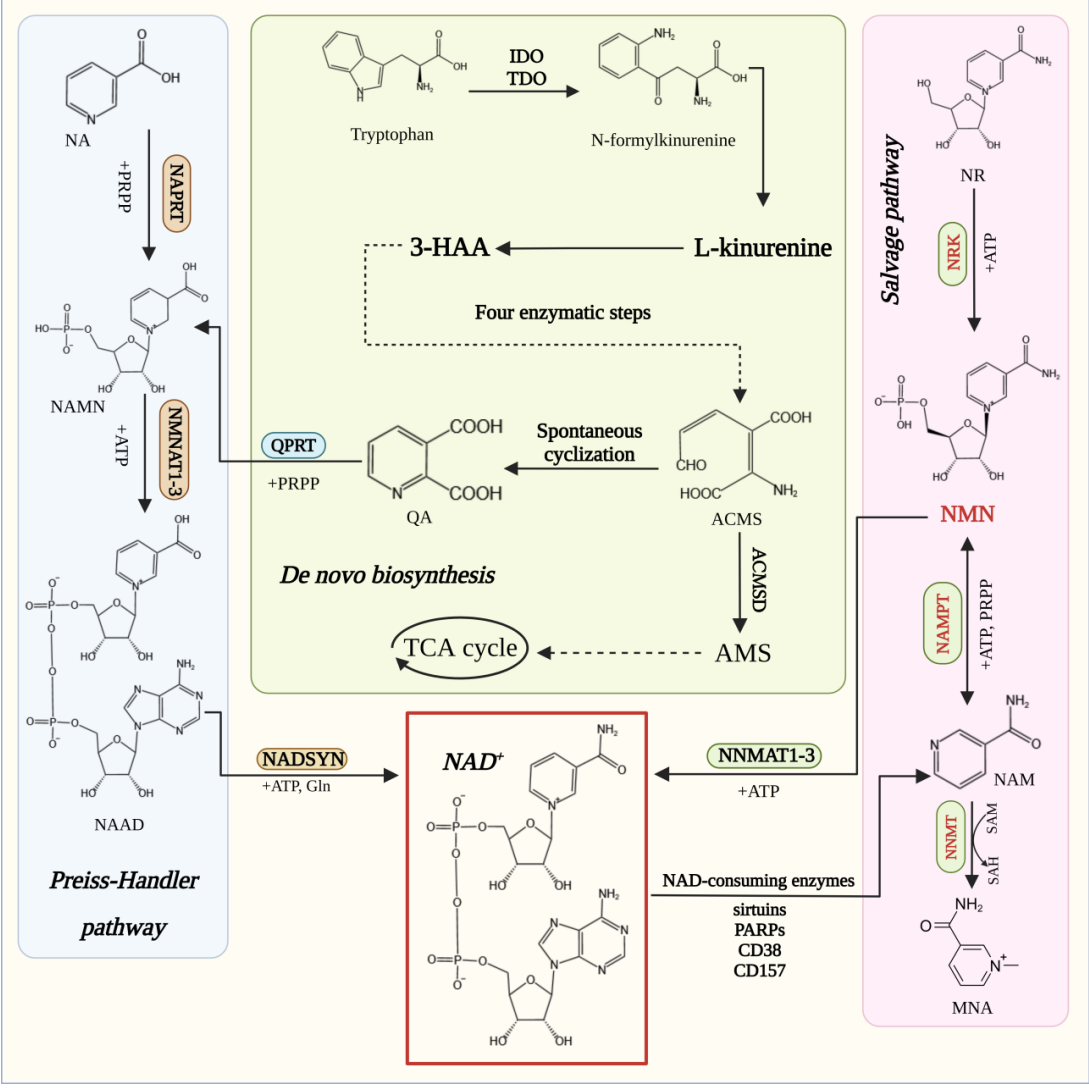
The common metabolic pathways of NAD^+^ and NMN. There are three major biosynthetic pathways of NAD+: the Preiss–Handler path-way (blue); the de novo synthesis pathway (green); and the salvage pathway (pink).

NMN is a direct precursor of nicotinamide adenine dinucleotide (NAD^+^). Accordingly, they share most of the metabolic pathways and co-mediate various cellular physiological metabolic functions (Figure 2) [5,6]. Studies have shown that NAD^+^ levels gradually decrease during aging in both rodents and humans, exacerbating aging-related diseases [7,8]. In 2016, Kathryn et al. [9] first found that NMN effectively alleviated age-related physiological decline in mice without any apparent toxicity, highlighting the tremendous potential of NMN supplementation as an effective anti-aging intervention in humans. Given the excellent efficacy of NMN in regulating physiological metabolism and promoting healthy aging, scientists have attempted to obtain NMR with high purity, high yield, and low cost through chemical catalytic synthesis and biosynthesis methods to meet the increasing market demand. To enable biosynthetic approaches, the pathways of intracellular NMN synthesis have been extensively studied [10,11,12]. Among them, the production of NMN from intracellular nicotinamide (NAM) under the catalysis of nicotinamide phosphoribosyltransferase (NAMPT) is the most important pathway, but it has a tight bottleneck due to limited NAMPT enzyme activity [13,14]. Nicotinamide N-methyltransferase (NNMT) does not directly participate in NMN synthesis, but it significantly impacts NMN homeostasis. By methylating nicotinamide to reduce its free content and affect the flux of NAM, an important precursor of the NMN salvage pathway, it regulates NAD^+^ levels. Thus, developing NNMT inhibitors could be a promising strategy to increase nicotinamide and NMN precursor levels [15,16]. In addition, nicotinamide nucleoside (NR) can also be converted into NMN under the catalysis of NR kinases NRK1 and NRK2 [17]. With the continuous clarification of the intracellular metabolic pathways of NMN, researchers have utilized synthetic biotechnology to transform key enzymes and metabolic pathways, producing NMN and related cellular proteins and providing sufficient raw materials for the application of NMN and its derivatives in fields such as nutrition, medicine, and animal husbandry.

Studies have shown that oral supplementation of NMN can rapidly increase the level of NAD^+^ in blood circulation, directly and indirectly affecting many key cellular functions, including metabolic pathways, DNA repair, chromatin remodeling, cellular aging, and immune cell function [1]. Importantly, NMN supplementation could also reverse the incidence of many aging-related diseases caused by the decline of NAD^+^ levels, including cognitive decline, cancer, metabolic diseases, osteoporosis, and frailty [18]. Targeting NAD^+^ metabolism by direct supplementation of NMN to ameliorate aging-related diseases and extend the healthy lifespan has become a potential approach for anti-aging and the regulation of metabolic imbalance [19]. This article reviews the main molecular strategies for promoting efficient NMN synthesis in chemical and biosynthetic processes from the perspectives of metabolic engineering, such as host screening and chassis cell optimization, as well as enzyme engineering approaches, such as gene mining and enzyme modification. In addition, the research progress on the regulatory effects of NMN on animal and human health is also reviewed. Finally, the future applications of artificial intelligence (AI) in improving the yield and efficiency of NMN synthesis, as well as the application of NMN products in the livestock and poultry industry, are also prospected, charting a path for the industrialization of NMN applications.

## 2. Chemical Catalytic Synthesis of NMN

The synthesis of NMN involves the chemical catalytic conversion of tetraacetyl ribose, benzoyl-β-D-ribose, and nicotinamide (NAM) as the main raw materials through ammonolysis, phosphorylation, glycosylation, and other chemical reactions. The three main synthetic routes are the bromoacetyl ribose method, trimethylsilyl trifluoromethanesulfonate (TMSOTf) catalytic condensation, and acid hydrolysis of AMP.

**Figure 3:**
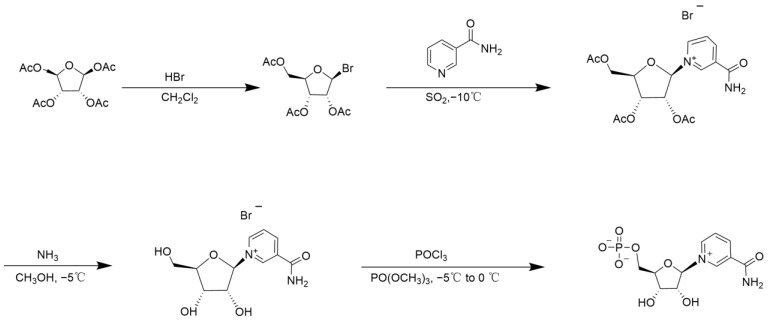
Bromoacetyl ribose method.

Ribose protected by acetyl or benzoyl groups can be brominated or chlorinated, and the protective group can then be removed through hydrolysis to yield NMN. Mikhailopulo et al. [20] utilized tribenzoyl-β-D-ribose as the starting material, which was first brominated with hydrobromide, and the bromine was subsequently replaced with nicotinamide. The benzoyl group was then eliminated under alkaline conditions, followed by phosphorylation using phosphorus trichloride/trimethyl phosphate to produce β-NMN. This method involves four steps and affords a total yield of approximately 31%. Based on the aforementioned route, Lee et al. [21] utilized tetraacetyl ribose and subjected it to bromination with hydrobromic acid, followed by nicotinamide substitution to generate nicotinamide triacetate. Subsequently, the acetyl group was eliminated using ammonia, and ultimately, β-NMN was obtained through phosphorylation with phosphorus oxychloride/trimethyl phosphate, followed by purification via resin chromatography. The overall yield amounted to approximately 57%, with purity exceeding 97% (Figure 3).

**Figure 4:**
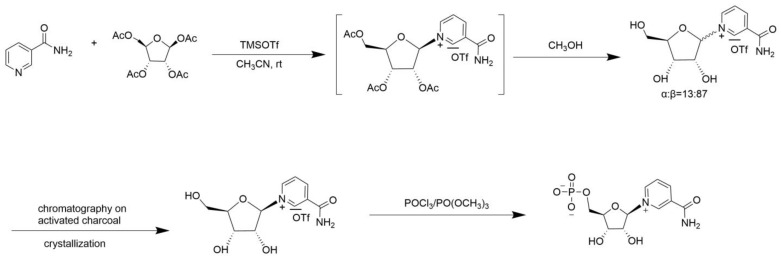
TMSOTf catalytic condensation.

**Figure 5:**
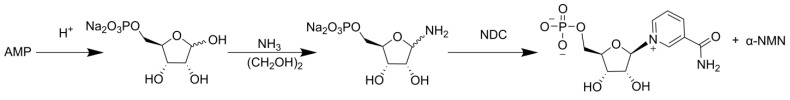
AMP acid hydrolysis method.

In the TMSOTf approach, nicotinamide is silanized and condensed with hydroxyl-protected ribose in the presence of TMSOTf, which generates the β-nucleoside with high stereoselectivity. Then, the nicotinamide is deprotected by NH_3_ or MeOH and hydrolyzed to NMN [22,23]. Compared with the previous approaches, this method shortened the reaction time and increased the stereoselectivity, while solvent utilization and product extraction were relatively simple, with a high yield of NMN (Figure 4). In the hydrolysis of adenosine monophosphate (AMP) under acidic conditions, ribose-5-phosphate is first formed, which is subsequently treated with anhydrous ammonia in dry ethylene glycol to yield ribose-5-phosphate amine. This intermediate compound is then reacted with l-(2,4-dinitrophenyl)-3-carbamoylpyridinium chloride (NDC) to produce NMN (α:β = 2:3) [24,25,26]. However, this method involves harsh reaction conditions and requires anhydrous intermediate steps, resulting in a higher proportion of the inactive α isomer (Figure 5).

Chemical catalysis methods offer advantages such as cost effectiveness, easy availability of raw materials, scalability, and high yields. Currently, industrial-scale production of NMN is predominantly achieved through chemical methods. However, chemical approaches come with drawbacks such as stringent reaction conditions, the use of organic solvents, and limited stereoselectivity. By contrast, biological synthesis pathways are characterized by high stereoselectivity, mild reaction conditions, and minimal by-products. These qualities make biological synthesis a primary area of research in the current exploration of NMN synthesis methods, offering a promising alternative to address the limitations of chemical approaches.

## 3. Biosynthesis of NMN

The biological synthesis pathway of NMN is closely related to the metabolic synthesis pathways of NAD^+^ in the organism. There are three major NAD^+^ synthesis pathways in mammals [27], including the de novo synthesis pathway, the Preiss–Handler pathway, and the salvage pathway, among which the latter is the key to maintaining normal NAD^+^ levels. According to the different substrates and enzymes required by each synthetic route, the salvage pathway is divided into the nicotinamide phosphoribosyltransferase (NAMPT) and nicotinamide riboside kinase (NRK) routes.

### 3.1. Enzymes Catalyzing the Synthesis of NMN

Nicotinamide phosphoribosyltransferase (NAMPT) catalyzes the synthesis of NMN from nicotinamide (NAM) and phosphoribosyl pyrophosphate (PRPP). In mammalian cells, NAMPT is the rate-limiting enzyme of the NAD^+^ metabolic pathway. Revollo et al. [28] discovered that NAMPT exhibited 46-fold lower efficiency compared to nicotinamide mononucleotide adenosine transferase (NMNAT), another enzyme involved in the salvage pathway, indicating that the reaction catalyzed by NAMPT represents the bottleneck of NAD^+^ synthesis from nicotinamide.

Marinescu et al. [29] individually expressed NAMPT orthologs from *Mus musculus*, *Shewanella oneidensis*, and *Haemophilus ducreyi* in *E. coli*. Compared to the two bacterial NAMPT enzymes, the mammalian NAMPT from *Mus musculus* exhibited a relatively lower yield of NMN in *E. coli*. Therefore, it is crucial to identify NAMPT variants with high enzyme activity for efficient industrial production of NMN by screening diverse sources. Shoji et al. [30] compared the heterologous expression of 10 different NAMPT genes from mammalian and bacterial sources in *E. coli* and found that the bacterial NAMPT gene from *Chitinophaga pinensis* exhibited the highest expression efficiency in *E. coli*, with a 2.4-fold higher enzyme activity than that of NAMPT from *S. oneidensis*. Similarly, Huang et al. [12] selected eight previously reported NAMPT genes from various sources for expression in *E. coli* and demonstrated that the NAMPT from *Vibrio* bacteriophage KVP40 was capable of intracellularly synthesizing 81.3 mg/L NMN using 200 mg/L nicotinamide as substrate, representing a significant increase in extracellular NMN accumulation.

Nicotinamide N-methyltransferase is a crucial cytosolic protein and a key methyltransferase. Inside cells, it catalyzes the transfer of a methyl group from the cofactor S-adenosyl methionine (SAM) to nicotinamide, leading to the generation of N1-methylnicotinamide (MNA) and S-adenosyl-L-homocysteine (SAH) [31,32]. In this case, NNMT exerts its influence on both the NAD^+^ salvage pathway and the methionine cycle by concurrently depleting both NAM and SAM. As a crucial precursor for NMN synthesis, NAM cannot replenish the NAD^+^ salvage pathway and regulate NAD^+^ levels upon methylation. Studies have demonstrated a significant impact of NNMT activity on cellular NAD^+^ content. Therefore, reducing NNMT activity using inhibitors represents a promising strategy to alleviate NAM depletion and enhance NAD^+^ availability [33,34,35]. NNMT has been demonstrated to play crucial and multifaceted roles in human health and disease. Aberrant NNMT expression has been observed in various diseases, including cancer [36,37], metabolic disorders [38], and neurodegenerative diseases [39]. Consequently, NNMT inhibitors have emerged as promising therapeutic agents for these diseases. Competitive inhibitors that displace SAM or nicotinamide are based on NNMT substrate binding competition. However, it is evident that targeting only one substrate pocket may not be sufficient for effective NNMT inhibition. Therefore, dual-target NNMT inhibitors have been developed and can bind to both substrate-binding pockets, thereby enhancing inhibitor potency and selectivity. However, the cellular and in vivo activity of these inhibitors is limited due to poor cellular uptake. Current research on dual-target inhibitors focuses on optimizing the structural features based on the functional groups of the NAM and SAM substrate-binding pockets [40,41,42,43,44]. Additionally, covalently binding compounds, natural products, and macrocyclic peptides can also serve as alternative NNMT inhibitors [45,46].

NMN can be synthesized by NRK from nicotinamide ribose, which is another substrate of the salvage pathway. Tao et al. [47] utilized NR as a direct substrate and ATP as a co-substrate to efficiently produce NMN through the catalytic action of *S. cerevisiae*-derived NRK, achieving a conversion rate of more than 90%, while the purity of NMN obtained after ion exchange chromatography and freeze drying exceeded 95%. Qian et al. [48] discovered a novel highly active NRK from *Kluyveromyces marxianus* (*Kma*NRK), exhibiting an unprecedented specific activity of 7.9 U/mg purified protein, surpassing all previously reported enzymes in this field. In the presence of an external ATP regeneration system (AcK/AcP), they achieved a molar isolation yield of 84.2% and a space–time yield of 281 g/L/day. In the human body, there are two highly conserved forms of NRK, designated NRK1 and NRK2, respectively [49,50]. Cheng et al. [51] used a semirational design to significantly improve the catalytic efficiency of the enzyme by introducing a beneficial substitution (G13S) near the ATP binding site of the human nicotinamide riboside kinase (HsNRK). As a result, a 100% conversion of 50 g/L NR was achieved.

### 3.2. Metabolic Engineering for NMN Synthesis

In addition to enhancing the enzyme activity of NMN synthase, optimizing the metabolic network of host strains is also a crucial approach for achieving efficient production of NMN. In the NMN synthesis pathway, one of the substrates of NAMPT, phosphoribosyl pyrophosphate (PRPP), is a key limiting factor affecting NMN production and is too expensive to add externally [52]. Therefore, how to accumulate a large amount of PRPP and thereby increase NMN production is a major issue that needs to be explored. On the other hand, the substrate of the NRK pathway, nicotinamide riboside, is also expensive and difficult to obtain, so its direct use as raw material is not feasible.

#### 3.2.1. Metabolic Engineering of the NAMPT Pathway

PRPP, the essential co-substrate for NMN biosynthesis, can be produced through the pentose phosphate pathway, utilizing 5-phosphoribosyl-1-phosphate as an intermediate. Consequently, the multienzyme cascade catalysis method offers a highly efficient and straightforward approach to NMN production [53]. Marinescu et al. [29] applied NAMPT orthologs from diverse sources for NMN synthesis in *E. coli* through fermentation. To enhance the supply of PRPP, they co-expressed ribose phosphate pyrophosphokinase (Prs) from *Bacillus amyloliquefaciens*, resulting in a remarkable fermentation yield of 15.42 mg/L. Black et al. [54] constructed three different metabolic pathways for NMN synthesis in *E. coli*, and the results showed that the overexpression of nicotinamide phosphoribosyltransferase (NadV) from *Ralstonia solanacearum* increased NMN yield 130 times compared with the starting strain, reaching 1.5 mmol/L.

Through metabolic engineering, the metabolic flux was enhanced to improve the yield of NMN from cost-effective substrates such as glucose, xylose, and ribose [11,53,55]. In addition, previous studies reported that native enzymes of *E. coli* can partially convert the product NMN into nicotinamide and other by-products. Therefore, the knockout of genes related to these pathways in host bacteria can block the conversion of the product and improve substrate utilization. Liu et al. [56] increased the expression of the endogenous *ygcS* gene in *E. coli* to enhance carbohydrate uptake while simultaneously knocking out genes encoding NMN aminohydrolase (*pncC*) and NMN adenylyltransferase (*nadR*). As a result, the highest yield of NMN was achieved, reaching 496.2 mg/L. The inhibitory effect of high substrate loading on the catalytic performance of the enzyme can be overcome by introducing an NMN transporter [51,57]. Shoji et al. [30] overexpressed the coding genes of seven enzymes in the pentose phosphate pathway (PPP) in *E. coli* (pgi, zwf, pgl, gnd, rpiA, rpiB, and prs) and identified two functional transporters (NiaP and PnuC). By enhancing the PRPP biosynthesis pathway and improving the transport, the efficient synthesis of NMN from extracellular glucose and nicotinamide was achieved, whereby a high NMN yield of 6.79 g/L was obtained in 8 h, with a substrate conversion rate of 86%. Building on this foundation, Huang et al. [12] employed a comprehensive metabolic engineering strategy, including the elimination of NMN degradation pathways, the selection of an optimal NAMPT enzyme, the introduction of nicotinamide transporters, the optimization of gene expression, and the optimization of culture conditions. Finally, 16.2 g/L NMN was obtained in a 5 L bioreactor.

#### 3.2.2. Metabolic Engineering of the NRK Pathway

Human NRKs were reported to have optimal pH values of 6.5–9.0, and they are very unstable at pH values outside of this range, as well as being sensitive to heat [58]. Immobilization is a commonly used strategy for improving the performance and robustness of enzymes. Moreover, immobilized enzymes can be recycled repeatedly in the production process, realizing an efficient and economical way to synthesize NMN. He et al. [59] expressed the NRK from *S. cerevisiae* at high levels in soluble form in *E. coli* and immobilized it using a metal affinity tag to optimize the enzyme performance. He et al. [60] expressed yeast-derived a-agglutinin anchoring protein Aga2 and human NRK-2 enzymes in tandem, which enabled NRK-2 to be efficiently displayed on the cell wall of *Saccharomyces cerevisiae*. Under optimized conditions, the substrate conversion rate reached 98.2%. NRK-2 showed good pH and heat stability, and it could be reused in industrial production. At present, NR substrates are generally synthesized by chemical methods and are expensive, which limits their large-scale industrial applications. After the study of NR as the core metabolic intermediate pathway, a variety of different substrate synthesis pathways were discovered. Zhao et al. [61] found that NRK was able to phosphorylate ribose-1-phosphate and ribose nicotinamide. Using ribose, NAM, and ATP as substrates, NMN was obtained via a one-pot reaction in a multienzyme catalytic system, with a substrate conversion rate of 92% after 4 h. Yu et al. [62] used adenosine and phosphate to generate NMN under the catalysis of purine–nucleoside phosphorylase (PNP) and NRK, to which they added polyphosphate kinase (PPK) derived from *Pseudomonas aeruginosa* to realize ATP recycling. Zhou et al. [11] designed the NR phosphorylation pathway to generate NMN, which could achieve an NMN yield of 3 g/L after screening and optimizing the multienzyme cascade catalytic system of nucleoside phosphorylase (PyNP), PNP, PPK2, and NRK with uridine as a substrate.

### 3.3. Protein Engineering of NMN Synthase

Highly active and robust NMN synthases are critical for the efficient and stable production of NMN. In addition to direct screening, another effective approach involves using computer-aided protein engineering to modify key enzymes. This method leverages computer simulations to quickly identify promising mutants through accurate predictions. Bioinformatic models are capable of predicting the active site of enzymes as well as assessing the impact of specific point mutations on enzyme stability, substrate affinity, and other functional aspects, facilitating precise enzyme modifications [63,64]. Peng et al. [65] developed a multienzyme cascade reaction and applied protein engineering to evolve crucial enzymes for the cost-effective in vitro synthesis of NMN from D-ribose, a readily available substrate. The research utilized tools, such as SWISS-MODEL and YASARA, for homology modeling and molecular docking of Cp-NAMPT, an essential enzyme in this pathway. Following established protocols, the team efficiently screened mutants using a high-throughput method based on substrate fluorescence [66]. The Cp-NAMPT mutant displayed a 57% increase in activity, significantly enhanced thermostability, and improved substrate binding compared to the wild-type enzyme. Substituting the wild type with the Cp-NAMPT-Y15S mutant increased the NMN titer from 3.96 g/L to 4.75 g/L. The targeted modifications of the key enzymes through protein engineering have markedly increased the efficiency of NMN production.

Du et al. [67] discovered two novel highly active NRKs from *Sparassis crispa* and *Beauveria bassiana*, with respective specific activities of 6.14 and 6.66 U/mg. *Beauveria bassiana* NRK (*Bba*NRK) was chosen for structural modification. Alphafold2 was utilized to model the structure of the identified wild-type *Bba*NRK, while AutoDock Vina was employed for substrate docking analysis. A total of eleven mutants were constructed and assessed, among which V172K (M1) exhibited 20% higher enzyme activity than the wild type. Then, the PLIP website was employed to calculate and analyze the binding affinity. Compared to the WT, M1 had a deeper substrate channel, enabling a shorter distance for phosphate-group transfer between substrates and bringing the γ-phosphate group of ATP closer to NR. In M1, residues K172, N92, and S93 established additional hydrogen bonds with the substrate. Notably, S93 directly stabilized the phosphate group of ATP, while residues R164 and Y171 engaged in π-bonding interactions with the substrate. This mutation resulted in a more stable and efficient binding conformation, significantly enhancing the enzyme’s catalytic efficiency. The lack of a solved NRK crystal structure poses challenges for 3D structural modeling and mutation site prediction. While directed evolution methods can modify proteins to some extent, they often lack precision and traceability. Deep learning and artificial intelligence (AI) techniques, trained on extensive sequence and structure datasets, have the potential to design protein molecules with entirely novel structures and molecular functions [68]. For instance, RFdiffusion, a protein design model grounded in diffusion models, can produce novel protein designs by iteratively refining protein structures while adhering to specific constraints [69]. Unlike traditional approaches, it does not rely on predefined templates or existing protein frameworks. Instead, it can directly generate desired protein structures starting from a random initial state, showcasing remarkable adaptability and innovation. Overall, AI models have significantly enhanced the success rate of de novo protein design, addressing crucial challenges, such as refining NRK structural nuances and ensuring precise complementarity with substrates.

### 3.4. Selection of NMN Synthesis Hosts

Currently, *E. coli* serves as the principal engineering strain for NMN biosynthesis research. As a prototypical model organism, *E. coli* possesses a well-defined genetic background and a plethora of gene editing tools, facilitating metabolic engineering modifications. Additionally, there are reports of utilizing alternative host strains for NMN synthesis. The food-grade expression system utilizing lactic acid bacteria, known for its efficiency and safety, has gained extensive attention in the food fermentation industry. Leveraging lactic acid bacteria as the host cells for NMN synthesis has emerged as a promising strategy. Sugiyama et al. [70] screened three candidates from a library of 174 strains of lactic acid bacteria, among which *Lactobacillus* RD011727 produced the highest NMN titer of 2 mg/L. Kong et al. [71] used CRISPR/nCas9 gene editing technology to knock out the *nadR* gene in *Lactococcus lactis* NZ9000 and heterologously expressed the nadE* gene from *Francisella tularensis*, resulting in a final intracellular NMN production of 2289 μmol/L/mg from 10 g/L of externally added NAM in 12 h. 

Previously, NMN synthesis was conducted in microorganisms using glucose as the carbon source. As a non-food feedstock, methanol holds great promise for bio-manufacturing from non-sugar carbon sources. The conversion of captured carbon dioxide into methanol through hydrogenation catalysis, followed by microbial conversion, presents an attractive avenue for low-carbon biosynthesis [72]. In recent years, some progress has been made in the research on NMN synthesis using *P. pastoris*, which expanded the substrate spectrum of NMN biosynthesis. Wang et al. [73] achieved secretory expression of the NRK enzyme in *P. pastoris* cells using the pPIC9K plasmid vector, with a crude extracellular enzyme preparation achieving a molar conversion rate of 92.6%. Li et al. [74] heterologously expressed the NAMPT enzyme and introduced NiaP and PnuC transport proteins to enhance substrate uptake and product excretion in *P. pastoris* cells, enabling whole-cell NMN synthesis and offering a new strategy for industrial production. Tian et al. [75] constructed the *Pichia pastoris* strain PC110-Δ0305 using the CRISPR-Cas9 gene editing system and heterologously expressed NAMPT in this strain for whole-cell biocatalytic synthesis of NMN. Methanol was added at 24 h intervals during the shake-flask fermentation stage to induce NMN synthesis. The intracellular NMN content reached a peak of 1.46 mg/g DCW after 24 h. Furthermore, the study established a high-fat diet (HFD) obesity model to investigate the anti-obesity effects of NMN. The results demonstrated that NMN supplementation modulated lipid metabolism, ameliorated obesity as well as lipid and glucose metabolism dysregulation, reduced hepatic fat accumulation, and mitigated liver injury in HFD mice. The study provides a theoretical basis for the production of NMN from methanol in *Pichia pastoris*, as well as its nutritional applications. Leveraging methanol as an abundant and cost-effective substrate, and *Pichia pastoris* can be engineered as a cellular factory for producing pure NMN or diversified products rich in NMN, with significant economic and environmental implications.

### 3.5. ATP Regeneration Systems

The synthesis of NMN by any biological enzyme requires a relatively costly substrate, ATP. Consequently, the ratio of ATP consumption to the production of NMN is crucial for the cost of the catalytic process. Therefore, the recycling of ATP, as well as the high activity and stability of each enzyme, are crucial for efficient and low-cost production of NMN. It has been reported that the introduction of an ATP regeneration system based on polyphosphate kinase (PPK) can significantly reduce ATP consumption, thereby reducing the cost of NMN production [76]. PPK facilitates the reversible transfer of a phosphate group between inorganic polyphosphate (PP) and ATP [77]. Given the abundance and low cost of PP, PPK-based ATP regeneration systems have garnered increasing interest. Li et al. [78] developed an artificial in vitro multienzyme cascade biocatalytic system and conducted an analysis of PPK/PP and PK/PEP regeneration modules [79]. The findings demonstrated that the PPK/PP module may not be an optimal choice for ATP regeneration in the biocatalytic NMN production system. The system utilizing PPK/PP exhibited extremely low NMN yields, which were attributed to the potent inhibitory effect of PP on ribose–phosphate diphosphokinase (PRS), hindering the conversion of ribose 5-phosphate (R5P) into the crucial substrate PRPP. However, engineering a PRS variant insensitive to PP inhibition could facilitate the introduction of cost-effective PP or pyrophosphate (PP_i_) into the biological system for ATP regeneration, thereby reducing costs. In the NAMPT-catalyzed NMN synthesis pathway, the substrate PRPP is converted into the by-product PP_i_, which promotes the hydrolysis of ATP and significantly enhances the substrate affinity of NAMPT, resulting in an 1100-fold increase in the catalytic efficiency of NMN synthesis [80]. Moreover, the addition of pyrophosphatase (PPase) to degrade PP_i_ into phosphate can increase the yield of NMN by approximately 50%. This enhancement may be attributed to the promotion of the reaction process through the degradation of the by-product [76].

## 4. NMN Regulates Physiological Metabolism

NMN is a direct precursor of nicotinamide adenine dinucleotide (NAD^+^) and plays a key regulatory role in more than 400 metabolic reactions [5]. It was reported that the level of NAD^+^ decreases with aging in mice and humans, as well as in models of Alzheimer’s disease and heart failure [81]. The depletion of NAD^+^ leads to a decrease in the activities of poly ADP-ribose polymerase (PARP) [82], sirtuins (SIRTs) [83], and the cluster of differentiation 38 (CD38) [84], which leads to a series of chain reactions, such as mitochondrial dysfunction, DNA repair dysfunction, and related degenerative changes. Several studies have shown that the supplementation of NMN can effectively ameliorate physiological metabolic disorders and related diseases caused by NAD^+^ deficiency.

### 4.1. Anti-Aging

With the aging of the global population, there is an increasing demand for anti-aging healthcare products with the function of prolonging the life span and delaying the onset of aging-related diseases [85,86,87]. As a precursor for the synthesis of NAD^+^, NMN slows the process of NAD^+^ depletion with age by increasing NAD^+^ levels in vivo and thus may serve as a potential anti-aging healthcare product [1,19,88]. In recent years, researchers have carried out extensive studies on the anti-aging effect of NMN in animal models and clinical trials (Table 1). A large body of evidence suggests that NMN has important beneficial effects in regulating physiological metabolism, such as protecting endothelial cells [89], improving blood supply [90], repairing metabolic dysfunction [91], and protecting the nervous system [92]. In 2021, researchers conducted a small randomized, double-blind clinical trial of 25 prediabetic postmenopausal women who were overweight or obese and received oral NMN (250 mg daily) for 10 weeks. The results showed that NMN increased muscle sensitivity to insulin in women in the treatment group compared with those in the placebo group [93]. In 2023, a similar study included 36 healthy adults (40–59 years, 38.9% male) who were treated with oral NMN (250 mg daily) for 12 weeks. The results showed that the serum levels of the NAD+ metabolite nicotinamide were significantly higher in the treatment group than in the placebo group, and the arterial stiffness index showed a decreasing trend.

### 4.2. Treatment of Metabolic Diseases

Obesity is increasing worldwide due to changes in diet and lifestyle [100]. Obese individuals are more likely to develop metabolic diseases, including cardiovascular disease [101], type 2 diabetes [102], non-alcoholic fatty liver disease [103], and arteriosclerosis [104]. In addition, studies found that obesity can exacerbate aging and shorten the healthy lifespan of the elderly [105]. There is a growing body of evidence that appropriate supplementation of NMN targeting the regulation of NAD^+^ metabolic balance has a significant therapeutic effect on obesity-related metabolic diseases and aging [5,18,106]. 

Initially, NAD^+^ was found to be involved in regulating the metabolic rate of yeast extracts, so the link between NAD^+^ and metabolism has been extensively studied [107,108]. NMN regulates metabolism by increasing NAD^+^ flux [7,19]. It is necessary to maintain the normal function of organs such as fat, muscle, intestines, kidneys, and liver. However, obesity, diabetes, and cardiovascular diseases caused by a high-fat diet will disrupt the metabolic balance, leading to a decrease in the level of NAD^+^ in tissues, reducing the activity of sirtuins, destroying mitochondrial function, and disrupting other NAD^+^-dependent cellular processes [7,8]. Numerous studies have shown that increasing intracellular NAD^+^ levels can drive the metabolic response of organisms towards equilibrium [109]. High levels of NAD^+^ were found to promote the deacetylase activity of nuclear SIRT1 and protect against metabolic diseases induced by a high-fat diet [81,91]. NMN can also regulate the NAD^+^ content of endothelial cells, inhibit endothelial inflammation, and improve NO-dependent functions. In this pathway, CD73 located on the luminal surface of endothelial cells converts NMN into extracellular nicotinamide riboside, which is an important protective mechanism for maintaining intracellular NAD^+^ [110]. 

NMN is the most effective and efficient precursor for improving the levels of NAD^+^ [7]. Mice treated with oral NMN exhibited a significant increase in NAD^+^ levels after only 15 min [5]. Multiple studies have demonstrated the potential of NMN supplements to restore diminished NAD^+^ levels associated with aging and mitigate obesity in rodent models [18,88]. This implies their potential therapeutic utility in reinstating metabolic health among obese individuals. While most relevant research focused on determining safe and efficacious NMN doses for elevating NAD^+^ levels in healthy subjects [111], recent clinical trials started exploring the efficacy of NAD^+^ precursors for enhancing metabolic health and glucose metabolism in obese patients.

### 4.3. Regulation of Immune Cell Function

Inflammation is considered a key driver of aging and related diseases, as well as an important risk factor for incidence and mortality in chronic diseases [112]. Chronic inflammation affects glucose and lipid metabolism as well as insulin sensitivity by affecting the interaction between immune cells and metabolically active muscle cells, stem cells, and adipocytes in tissues and organs, thus exerting a systemic impact on the whole body’s metabolism [113,114]. Macrophage polarization is present in the visceral tissues of both elderly and obese individuals, accompanied by the enhanced expression of pro-inflammatory cytokines, such as tumor necrosis factor, IL-6, and IL-1β, as well as insulin resistance and a decreased rate of lipolysis [115,116]. The upregulation of NAD^+^ biosynthesis or degradation pathways regulates the early activity of macrophages and influences their activation [117]. Pro-inflammatory M1-like macrophage polarization enhances CD38 expression and increases NAD^+^ depletion. Conversely, research has linked the polarization of anti-inflammatory M2-like macrophages to elevated NAD^+^ levels dependent on NAMPT [118,119]. Inhibiting the NAM salvage pathway notably decreased the expression of certain genes linked to the distinctive phenotypes of both M1 and M2 macrophages [120]. Remarkably, this impact was counteracted by augmenting NAD^+^ levels through supplementation, which bypasses NAMPT inhibition [121]. The important role of NMN in rescuing the biological processes of macrophage activation suggests that as a key metabolite of macrophages, it is involved in the regulation of macrophage polarization and pro-inflammatory responses [122].

Chronic inflammation is associated with changes in the function of immune cells and a reduction in their ability to respond [119]. Aging is also an important factor, leading to imbalance or skewing of immune cell populations, including decreased levels of T and B cells, as well as a loss of T cell antigen receptor diversity [119]. Studies have shown that intracellular NAD^+^ and NAD^+^-depleting enzymes play roles in regulating the biological functions of T cells, including signals that can induce cell death in specific T-cell subsets, affecting T-cell polarization [123]. Metabolic reprogramming of immune cells can affect the age-related dysfunction of adaptive immunity by manipulating the NAD^+^-associated pathways, for example, by inhibiting CD38 to upregulate the NAD^+^-SIRT1-FOXO1 axis and increase T-effector cell function [115]. Thus, targeting NAD^+^ metabolic pathways using CD38 inhibitors or NAD^+^ precursors, such as NMN, to modulate cellular metabolic reprogramming is a potential therapeutic strategy to improve the function of the adaptive immune system in the elderly in the future.

### 4.4. Effects on Neurodegeneration

Older mammals have lower levels of NAD^+^ in brain cells, which is correlated with a higher incidence of neurodegeneration [124]. Although it has been observed in age-related neurodegenerative diseases, including Alzheimer’s disease [125], Parkinson’s disease [126], and amyotrophic lateral sclerosis (ALS) [127], the underlying causes and mechanisms of NAD^+^ loss in brain cells in these patients remain largely unknown. Various lines of evidence support a neuroprotective effect of NAD^+^ [128,129]. Rapid depletion of NAD^+^ has been found to be a major contributor to axonal degeneration and neuronal derangement [130]. During axonal degeneration, the transport of nicotinamide mononucleotide adenosyltransferase 2 (NMNAT2) along axons is hindered, resulting in rapid degradation of the axonal pool of this protein, leading to significant depletion of NAD^+^ levels within axons [131]. Conversely, axonal injury activates the NAD^+^-depleting enzyme SARM1, which facilitates axonal degradation by accelerating NAD^+^ breakdown [132]. The role of NMNAT2 and SARM1 in axonal injury has been demonstrated in several models [133]. In mice, Sarm1 knockout can prevent axonal degeneration and rescue severe axonal growth defects as well as related diseases caused by the lack of NMNAT2 [134].

Furthermore, it has been reported that the expression of the NAD^+^-depleting enzyme CD38 is increased during the development of Alzheimer’s disease. Conversely, NAD^+^ levels were found to be elevated in the brains of a CD38 knockout mouse model, which also showed a milder disease phenotype [135]. Pro-inflammatory cytokines also induce high levels of CD38 in microglia, astrocytes, neurons, and endothelial cells in the brain [136]. These findings consistently show that CD38 expression is associated with high levels of pro-inflammatory microglia and neuroinflammation in the mouse brain. In addition, activation of the NAD^+^-depleting enzyme PARP1 is also involved in the pathogenesis of Alzheimer’s and Parkinson’s disease [136,137]. In vivo studies using various models of these diseases have shown that the knockdown of PARP1 protects the brain from dysfunction and cognitive decline [138]. As a central metabolite for maintaining a healthy nervous system, NAD^+^ can, therefore, affect the biological function of various brain cell types [139,140]. Accordingly, counteracting the decline of NAD^+^ levels associated with aging may be an effective strategy for treating neurodegeneration. It is noteworthy that NMN supplementation to restore NAD^+^ levels resulted in demonstrable improvements in neural cell health, memory, and cognitive function in rat and mouse models of Alzheimer’s disease [141,142]. NMN treatment also exhibited neuroprotective effects in a *Drosophila* model of Parkinson’s disease and a mouse model of amyotrophic lateral sclerosis (ALS) [5]. Importantly, clinical trials also tested NMN for the treatment of neurological diseases and the promotion of healthy aging [111]. These experiments will undoubtedly expand our understanding of NMN/NAD^+^ metabolism during human neurodegeneration.

### 4.5. Other Diseases

**Figure 6:**
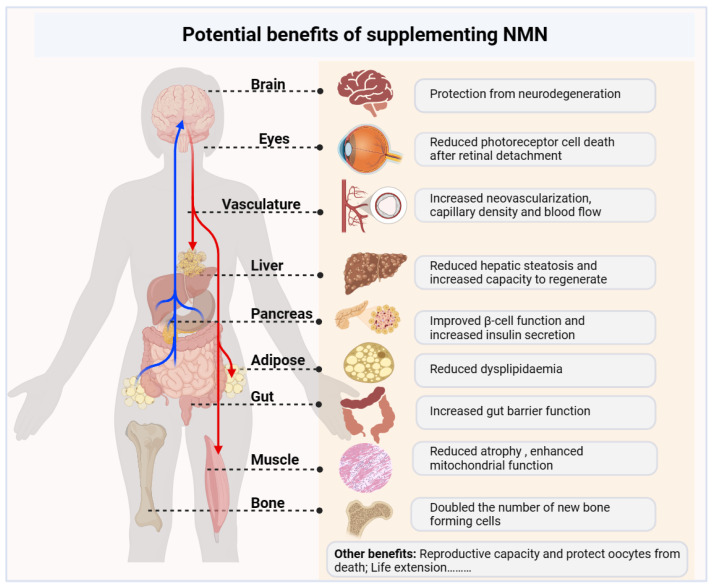
Potential benefits of NMN supplementation. As a supplemental precursor of NAD+, NMN has a modulating effect on the aging-related deterioration of physical function or diseases, including preventing cognitive decline, improving tissue and organ function, improving metabolic health, reducing inflammation, and increasing physiological benefits, which together may extend a patient’s healthy and overall lifespan.

Restoring NAD^+^ levels through supplementation with NMN has become a therapeutic approach for the prevention and treatment of various aging-related diseases, with the aim of restoring health and vitality in the elderly (Figure 6). In a mouse model of carotid artery occlusion, exogenous NMN supplementation reduced retinal inflammation and photoreceptor death while activating antioxidant genes and reducing retinal dysfunction in the early retinal detachment phase [143]. NMN can also reduce corneal inflammation caused by hypertonic tears at the early stage of dry eye by regulating the interaction between corneal epithelial cells and macrophages [144]. In a mouse model of acute kidney injury (AKI), NMN treatment significantly reduced renal tubular injury, cellular aging, and renal fibrosis [145]. In another study, NMN was found to increase the levels of NAD^+^ and reduce oxidative stress and barrier dysfunction in the gut of aging mice [146]. Similarly, NMN treatment reduced ethanol-induced liver injury in mice by increasing NAD^+^ levels, restoring the ERK1/2 pathway, or blocking the overexpression of ATF3 [147]. In summary, NMN seems to indirectly promote healthy aging and ameliorate related disease states by directly increasing NAD^+^ levels (Table 2).

## 5. Conclusions and Outlook

NMN is considered the best supplementary precursor of NAD^+^, and its biological activity is largely the result of its efficient conversion into NAD^+^, which acts as a fulcrum of energy metabolism and is involved in many physiological processes [88,108]. As a consequence, it affects mitochondrial function, the tricarboxylic acid cycle, lipid metabolism, and DNA damage repair [81]. So far, direct supplementation with NMN has been found to alleviate inadequate levels of NAD^+^ in organisms under specific conditions such as aging, obesity, and metabolic disorders, which has been confirmed by numerous experimental and clinical studies in cells, animals, and human patients [164]. As a consequence, NMN has been applied in health products, cosmetics, and food additives. With the increasing market demand for NMN and related products, high-yield, low-cost, safe, and environmentally friendly NMN synthesis methods have become an important problem in need of a solution. Although the physiological metabolic regulation function of NMN and research on its mechanism have been widely reported, excessive intake of NMN may cause side effects, such as cellular metabolic disorders and oxidative stress [7]. Animal studies have shown that high-dose NMN supplementation may affect blood sugar metabolism, cause weight gain, or change lipid metabolism [165]. Preliminary clinical trials have evaluated high-dose intake of NMN. Although no serious side effects were found in the short term, long-term safety needs further verification. Hence, most of the existing studies focused on the effects of NMN supplementation at low to medium doses. Research on the potential side effects of high-dose NMN is still in its infancy, and more long-term studies are needed to evaluate its long-term safety.

While the chemical synthesis of NMN has been industrialized, with easily controllable process technology, challenges arise from excessive impurities and the presence of chiral by-products, causing difficulties in separation and resulting in a diminished overall yield. Moreover, the substantial use of organic solvents raises concerns of environmental pollution that cannot be overlooked. In response to these challenges, the current focus of research lies in the biological synthesis of NMN. These pathways include a sequence of enzymatic reactions involving nicotinamide, nicotinamide ribose, and ATP. Biocatalysis offers the advantages of environmental safety and high substrate conversion rates with almost no impurities. However, obstacles include the cost and difficulty of obtaining NR and PRPP as substrates. Additionally, another significant hurdle is the substantial consumption of ATP in the enzymatic catalysis process, leading to elevated costs of biosynthesis that still prohibit its large-scale industrial application.

Utilizing synthetic biology strategies, significant progress has been made in metabolic engineering for the enhancement of NMN synthesis. This includes screening and modifying exogenous, highly active NMN synthases, increasing the accumulation of substrates, improving NMN transport efficiency, and knocking out genes related to NMN degradation. Despite significant progress, there is still room for improvement in the productivity of NMN synthesis. For example, studies on NMN biosynthesis mostly used *E. coli* as the engineering chassis and only analyzed the titer of the final product, with few studies focusing on improving the ATP supply system. Therefore, strategies that should be tested in the future include other excellent chassis cells and selecting different promoters for expression, detecting the concentration of the intermediate products during the reaction process to determine the limiting step of the multienzyme pathway, introducing a recycling system to optimize the supply of ATP, and designing different synthetic routes from relatively cheap and simple raw materials. If a high conversion rate is achieved and the overall cost of the whole production process is reduced, NMN biosynthesis can become economically competitive. Taken together, these biosynthetic strategies offer additional possibilities for obtaining high-quality NMN raw materials and also lay the foundation for expanding the range of applications of NMN in the prevention and treatment of a number of increasingly prevalent age-related diseases.

## Figures and Tables

**Table 1:** The metabolic effects of NMN in mouse models and human clinical trials.

Human Clinical Trials Focusing on Ageing			
Compound	Study Design	Dose and Duration	NcT/uMiN No.
NMN	Healthy volunteers aged from 40 to 65 years	Oral administration Long-term NMN administration 300 mg daily for 60 days	NCT04228640
	Healthy male volunteers aged from 40 to 60 years	Oral administration Long-term NMN administration for 8 weeks Dose is not described	UMIN000030609
	Double-blind study in postmenopausal and prediabetic women aged 55–75 years	Oral administration Long-term NMN administration: 250 mg daily for 8 weeks	NCT03151239
	Healthy male volunteers aged from 40 to 60 years	Oral administration Single administration of 100, 250, or 500 mg NMN	UMIN000021309
	Healthy volunteers aged from 50 to 70 years	Oral administration Long-term NMN administration: 100 mg or 200 mg for 24 weeks	UMIN000025739
Mouse models focusing on metabolism			
Compound	Model	Results	References
NMN	Tg (SIRT2) and Bubr1H/^+^ mice	Increased NAD^+^ contents and restored BubR1 levels	[94]
	Male C57BL/6N mice	Inhibited age-induced weight gain, increased insulin sensitivity, plasma lipid levels, physical activity, and energy expenditure, while also improving mitochondrial function in muscles	[1,9]
	p32cKO mice	Enhanced skeletal muscle mitochondrial oxidative metabolism in aged mice	[95]
	Male Long–Evans rats, ischemia–reperfusion or cisplatin-induced acute kidney injury in Sirt1^+/−^ mice, C57BL/6 mice, and 129S2/Sv mice	Improved mitochondrial function, decreased inflammation, improved physiological reserve, and decreased mortality, despite having no major effects on blood pressure or oxidative damage. Protected renal function from cisplatin-induced injury in wild-type but not Sirt1^+/−^ mice	[1,96]
	High-fat diet-induced obese female mice	Improved glucose tolerance and increased liver citrate synthase activity, and triglyceride accumulation	[97]
	Transverse aortic constriction-stressed mice, male conditional knockout mice, male cardiac-specific Fxn-knockout mice (Friedreich ataxia cardiomyopathy model), and male Sirt3-knockout/Fkn-knockout mice	Improved mitochondrial function and protection from heart failure. Improved cardiac function, reduced energy waste, and improved energy utilization in Fxn-knockout mice but not in Sirt3/Fkn double-knockout mice	[98,99]

**Table 2:** Recent progress on the regulation of physiological metabolism by NMN.

Research Field	Research Progress	Reference
DNA repair	NMN increased the telomere length of liver cells in fibrotic mice. NMN activated DNA repair proteins, such as PARP1, in aged mice.	[148] [149]
Metabolism	NMN increased insulin sensitivity in mice and humans. NMN increased the number of mitochondria in the liver of obese mice. NMN enhanced the efficiency of energy production in mouse mitochondria.	[150] [151] [152]
Cancer	NMN enhanced the efficacy of PD-1-mediated immunotherapy. NMN improved the cognitive function of mice after chemotherapy.	[153] [154]
Bone repair	NMN doubled the number of new bone-forming cells in mice.	[155]
Cardiovascular	NMN reversed the decline of vascular elasticity in aged mice.	[156]
Eye function	NMN reduced photoreceptor cell death after retinal detachment in mice.	[157]
Immunity	NMN enhanced the action of immune T cells and stimulated the production of immunoglobulins.	[158]
Lifespan	NMN prolonged the lifespan of mice.	[9] [146]
Nervous system	NMN improved cognition and memory in a rodent model of Alzheimer’s disease. NMN ameliorated depressive behavior in model mice.	[142] [159]
Reproductive system	NMN improved the reproductive ability of female mice. NMN protected porcine oocytes from cell death due to exposure to environmental toxins.	[160]
Skin and muscles	NMN promoted muscle remodeling in older individuals.	[161]
Organ health	NMN reduced liver damage in chronic alcohol intake model mice, reduced renal cell death, and protected the kidneys from ischemic damage. NMN promoted the development and differentiation of intestinal stem cells in mice.	[162] [163]

## Data Availability

This manuscript is entirely the author’s original work, with no paragraphs copied from other sources and no artificial intelligence tools used in its writing or generation. All data, analysis, and conclusions are based on the author’s original research and independent thinking.

## Abbreviation

NA: nicotinic acid; NAPRT, nicotinic acid phosphoribosyltransferase; NAMN, nicotinic acid mononucleotide; NMNAT, NAMN transferase; NAAD, nicotinic acid adenine dinucleotide; NADSYN, NAD^+^ synthase; IDO, indoleamine 2,3-dioxygenase; TDO, tryptophan 2,3-dioxygenase; 3-HAA, 3-hydroxyanthranilic acid; ACMS, α-amino-β-carboxymuconate-ε-semialdehyde; QA, quinolinic acid; QPRT, quinolinate-phosphoribosyltransferase; ACMSD, ACMS decarboxylase; AMS, α-amino-β-muconate-ε-semialdehyde; NR, nicotinamide riboside; NRK, nicotinamide riboside kinase; NAM, nicotinamide; NAMPT, nicotinamide phosphoribosyltransferase; NMN, nicotinamide mononucleotide; NMNAT, nicotinamide mononucleotide adenylyltransferase; NNMT, nicotinamide N-methyltransferase; SAM, S-adenosyl-L-methionine; SAH, S-adenosyl-L-homocysteine; MNA, 1-methyl nicotinamide; and NAD^+^, nicotinamide adenine dinucleotide
